# Grover’s algorithm in a four-qubit silicon processor above the fault-tolerant threshold

**DOI:** 10.1038/s41565-024-01853-5

**Published:** 2025-02-20

**Authors:** I. Thorvaldson, D. Poulos, C. M. Moehle, S. H. Misha, H. Edlbauer, J. Reiner, H. Geng, B. Voisin, M. T. Jones, M. B. Donnelly, L. F. Peña, C. D. Hill, C. R. Myers, J. G. Keizer, Y. Chung, S. K. Gorman, L. Kranz, M. Y. Simmons

**Affiliations:** 1 Silicon Quantum Computing Pty Ltd, UNSW Sydney, Sydney, New South Wales Australia; 2 Centre of Excellence for Quantum Computation and Communication Technology, UNSW Sydney, Sydney, New South Wales Australia

**Keywords:** Qubits, Quantum dots, Quantum information

## Abstract

Spin qubits in silicon are strong contenders for the realization of a practical quantum computer, having demonstrated single- and two-qubit gates with fidelities above the fault-tolerant threshold, and entanglement of three qubits. However, maintaining high-fidelity operations while increasing the qubit count remains challenging and therefore only two-qubit algorithms have been executed. Here we utilize a four-qubit silicon processor with all control fidelities above the fault-tolerant threshold and demonstrate a three-qubit Grover’s search algorithm with a ~95% probability of finding the marked state. Our processor is made of three phosphorus atoms precision-patterned into isotopically pure silicon, which localise one electron. The long coherence times of the qubits enable single-qubit fidelities above 99.9% for all qubits. Moreover, the efficient single-pulse multi-qubit operations enabled by the electron–nuclear hyperfine interaction facilitate controlled-Z gates between all pairs of nuclear spins with fidelities above 99% when using the electron as an ancilla. These control fidelities, combined with high-fidelity non-demolition readout of all nuclear spins, allow the creation of a three-qubit Greenberger–Horne–Zeilinger state with 96.2% fidelity. Looking ahead, coupling neighbouring nuclear spin registers, as the one shown here, via electron–electron exchange may enable larger, fault-tolerant quantum processors.

## Abstract (web summary)

A four-qubit processor of three phosphorus nuclear spins and an electron spin in silicon enables the implementation of a three-qubit Grover’s search algorithm with 95% fidelity. The implementation is based on an advanced multi-qubit gate with single-qubit gate fidelities above 99.9% and two-qubit gate fidelities above 99%.

## Main

Spin qubits in silicon hold great promise for the realization of large-scale quantum computers owing to their long coherence times, compatibility with advanced manufacturing technology and the possibility to operate at elevated (~1 K) temperatures^1–4^. However, correcting for unavoidable errors requires large numbers of qubits with sufficient quality. The well-known surface code^5,6^ demands that the fidelity of every qubit operation within these multi-qubit processors (initialization, readout and single- and two-qubit control) is above a threshold of approximately 99%. Although high-fidelity initialization, readout and single- and two-qubit gates have been demonstrated in gate-defined quantum dots^7–14^, combining all of these operations within a single multi-qubit device remains challenging. Reports on the successful implementation of multi-qubit algorithms therefore remain scarce (see Supplementary Table 1 in Supplementary Section I). First results on two-qubit algorithms^15,16^ have recently been followed by the implementation of quantum algorithms in two-qubit processors with single- and two-qubit gate fidelities above 99% (refs. ^11,12^). While coherent operations have been demonstrated in larger processors (three to six qubits)^17–19^, only a three-qubit phase-flip quantum error correction code has been executed for devices in which just single-qubit gate fidelities were reported above the fault-tolerant threshold^20,21^.

Phosphorus (^31^P) atom qubits in silicon (Si) have a number of unique and beneficial properties that can help overcome the challenges of implementing multi-qubit algorithms. The strong natural confinement of atom-based processors allows the exploitation of the hyperfine interaction between the phosphorus nuclear spins and the bound electron spin. This allows individual qubit addressability^22,23^, while also providing all-to-all qubit connectivity in the form of efficient multi-qubit gates. The latter leverages the fact that a single gate on the electron spin can entangle multiple nuclear spins. This reduces the number of operations needed to execute quantum algorithms. In addition, the nuclear spins have long coherence times^24^ and can be read out with high fidelity via the process of quantum non-demolition readout^25^. Phosphorus atom qubits in silicon have been realized using ion implantation with high-fidelity single- and two-qubit gate operations and the recent demonstration of entanglement between two nuclear spins and one electron spin^23,26^. Scaling to larger error-corrected architectures requires precision control over the placement of the phosphorus atom qubits, which can be achieved by scanning tunnelling microscopy (STM) lithography^27,28^.

Here, we demonstrate full coherent control over a precision-manufactured four-qubit processor in Si defined by three phosphorus nuclear spin qubits and one electron spin qubit. We achieve single-qubit gate fidelities for all four individual qubits of (99.94 ± 0.01)%, (99.98 ± 0.01)%, (99.95 ± 0.01)% and (99.95 ± 0.01)%. In addition, we demonstrate two-qubit controlled-*Z* (CZ) gates between all pairs of nuclear spins with fidelities of (99.65 ± 0.35)%, (99.49 ± 0.39)% and (99.32 ± 0.22)%, as well as readout of all nuclear spin qubits with a fidelity above 99% after post-selection. We exploit these high-fidelity (>99%) operations, all achieved within the same device, and the all-to-all qubit connectivity in the processor to produce Bell states and a three-qubit Greenberger–Horne–Zeilinger (GHZ) state with fidelities above 96%. Finally, we benchmark our four-qubit processor by executing a Grover’s search algorithm on the three nuclear spin qubits with a (94.57 ± 2.63)% average success probability of finding the marked state compared with the ideal algorithm fidelity. This constitutes one of the most successful implementations of this algorithm in any qubit platform so far.

## Single-qubit operations

**Fig. 1:**
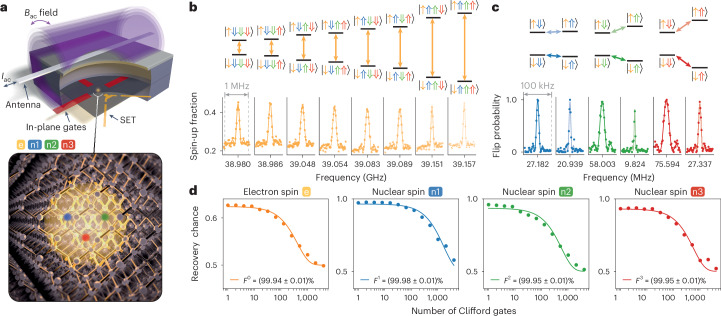
Single-qubit operations in the four-qubit processor. **a**, Top: a schematic illustration of the device consisting of in-plane gates (red), a SET charge sensor (yellow) and an antenna (light-grey wire). By applying an alternating current *I*_ac_ through the antenna, an alternating magnetic field *B*_ac_ is produced, enabling magnetic control. Bottom: an artistic impression of the three phosphorus atoms (blue, green and red) incorporated into the silicon crystal. The attracted electron wave function is depicted in yellow. The three nuclear spins and the electron spin define the four-qubit quantum processor. **b**, Measured ESR spectrum displaying eight resonance frequencies (bottom), each corresponding to a different nuclear spin configuration (top; see energy level diagram). Lighter colours indicate higher resonance frequency. **c**, Measured NMR spectrum (bottom) showing all six electron spin state-controlled peaks (top; see energy level diagram). Lighter colours correspond to transitions with electron-$\left\vert \uparrow \right\rangle$, and darker colours to electron-$\left\vert \downarrow \right\rangle$. **d**, RB decay curves for all four qubits, annotated with the corresponding average physical gate fidelity calculated from the measured Clifford fidelity and the error obtained from the fit. All single-qubit control fidelities surpass 99.9% fidelity.

The multi-qubit processor studied in this work is formed by patterning three ^31^P atoms into isotopically purified ^28^Si with atomic precision using STM hydrogen lithography, as previously described for the same device by Reiner et. al.^28^. Highly phosphorus-doped silicon in-plane gates allow control of the electrostatic environment of the P atoms (highlighted in red in the schematic illustration of the device in Fig. 1a, top). An electron can be loaded onto the P atoms from a nearby, tunnel-coupled single-electron transistor (SET, yellow) that also serves as a charge sensor. To control the nuclear spins (basis states $\left\vert \Downarrow \right\rangle$, $\left\vert \Uparrow \right\rangle$) and the electron spin (basis states $\left\vert \downarrow \right\rangle$, $\left\vert \uparrow \right\rangle$), a broadband antenna (grey) is placed on top of the device, delivering the radiofrequency and microwave signals for nuclear magnetic resonance (NMR) and for electron spin resonance (ESR), respectively. An artistic impression of the four-qubit processor is shown in Fig. 1a (bottom), where the electron wave function (yellow) spreads over the three P atoms (blue, green and red) that are embedded in the silicon crystal. Henceforth, we use label 0 for the electron spin qubit and labels 1, 2 and 3 for the nuclear spin qubits (blue, green and red, respectively).

Electron spin initialization and readout is performed via a ramped technique^29^ at a dilution refrigerator base temperature of 15 mK, with an applied magnetic field of 1.45 T. Quantum non-demolition readout of the nuclear spins is achieved with fidelities above 99% after post-selection as shown in Supplementary Section II with nuclear spin initialization shown in Supplementary Section III.

When an electron is loaded onto the multi-nuclear spin register, the electron spin interacts with all the nuclear spins through the contact hyperfine interaction, causing the ESR frequency to depend on the state of each of the nuclear spins. Figure 1b (bottom) shows an ESR spectrum with eight resonance peaks, where each peak corresponds to a different configuration of the nuclear spins (for the energy level diagram, see Fig. 1b, top). From the ESR peak separations, we find hyperfine interaction strengths of *A*_1_ = 6 MHz, *A*_2_ = 68 MHz and *A*_3_ = 103 MHz. The presence of the hyperfine interaction also allows each nuclear spin to be addressed separately, with the NMR frequency depending on the targeted nuclear spin and the state of the electron spin. An NMR spectrum displaying all six expected peaks is shown in Fig. 1c (bottom; for the energy level diagram, see Fig. 1c, top).

Having established full individual addressability of the electron spin and the three nuclear spins, we measure the dephasing time of each qubit using a Ramsey experiment. We find ${T}_{2}^{\,* }=28.1\,\upmu {\rm{s}}$ for the electron spin and 1.26 ms, 0.49 ms and 0.60 ms for nuclear spins 1, 2 and 3, respectively (Supplementary Section IV). To measure the dephasing time of the electron spin, we initialize all nuclear spins into the $\left\vert \Downarrow \Downarrow \Downarrow \right\rangle$ state before applying the ESR pulses conditional on that nuclear spin configuration (for all other nuclear spin configurations we find similar ${T}_{2}^{\,* }$ values; Supplementary Section IV). Based on these dephasing times, Rabi dephasing times and the Rabi frequencies (also shown in Supplementary Section IV), we obtain qubit quality factors $Q={T}_{2}^{\,* } {f}_{{\rm{Rabi}}}$ of 4.82, 14.14, 11.84 and 18.86, as well as gate quality factors of $Q={T}_{2}^{\,{\rm{Rabi}}} {f}_{{\rm{Rabi}}}$ of 34, 797, 84 and 69, for the electron spin, nuclear spin 1, nuclear spin 2 and nuclear spin 3, respectively.

Next, we characterize the control fidelity of all single-qubit operations by means of randomized benchmarking (RB). We achieve physical gate fidelities, *F*^*i*^, above 99.9% for all four qubits (*i* = 0, 1, 2 or 3) as displayed in Fig. 1d. RB for the electron spin is performed with the nuclear spins initialized into the $\left\vert \Downarrow \Downarrow \Downarrow \right\rangle$ state (all other nuclear spin configurations also yield fidelities above 99.9%; Supplementary Section V), while RB for the nuclear spins is performed with the electron spin initialized into the $\left\vert \downarrow \right\rangle$ state. With high-fidelity nuclear spin readout and all single-qubit gate fidelities surpassing the fault-tolerant threshold, we proceed to create entanglement between the nuclear spins.

## Two- and three-qubit entanglement

To entangle two of the nuclear spins, we exploit the hyperfine interaction between the electron spin and each of the nuclear spins. Here, simply by enacting a 2π rotation of the electron spin conditional on the configuration of the nuclear spins, we implement a geometric CZ gate between the nuclear spins^23,30^. To illustrate this, starting with the control nuclear spin in state $\left\vert \Downarrow \right\rangle$, the target nuclear spin in $(\left\vert \Downarrow \right\rangle +\left\vert \Uparrow \right\rangle )/\sqrt{2}$ and the third nuclear spin in $\left\vert \Downarrow \right\rangle$, an ESR 2π pulse conditional on $\left\vert \Downarrow \Downarrow \Downarrow \right\rangle$ flips the target nuclear spin by 180° around the *z* axis of its Bloch sphere. If, however, the control state was in state $\left\vert \Uparrow \right\rangle$, the same ESR pulse would not affect the target nuclear spin. To implement this gate irrespective of the state of the third nuclear spin, an additional ESR 2π pulse conditional on $\left\vert \Downarrow \Downarrow \Uparrow \right\rangle$ can be applied, creating a two-qubit CZ gate. Inserting the two ESR pulses in between a −π/2 rotation and a π/2 rotation of the target nuclear spin results in a nuclear controlled-NOT gate.

**Fig. 2:**
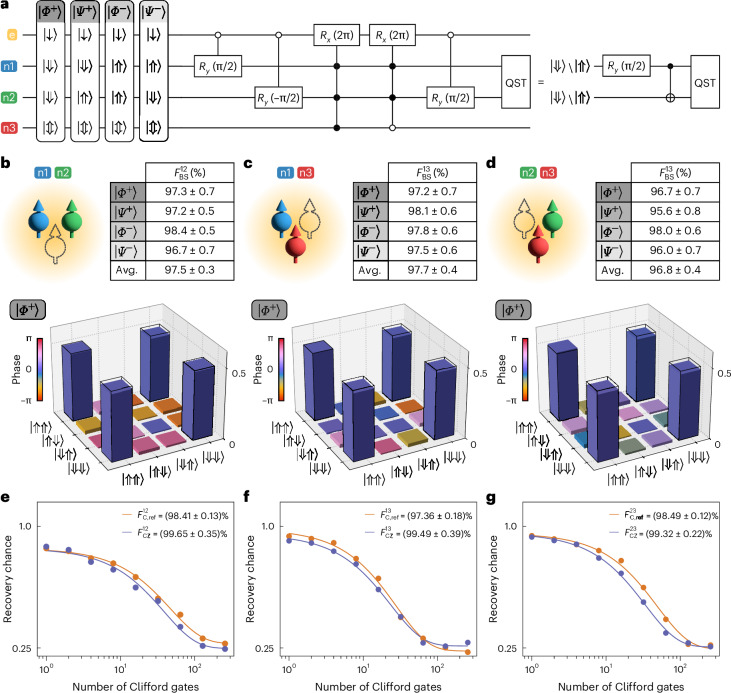
Bell state tomography and two-qubit RB. **a**, The circuit diagram used to construct the Bell states for nuclear spins 1 and 2. The table presents the input states corresponding to the final Bell state. **b**–**d**, Fidelities for all Bell states and reconstructed density matrices for $\left\vert {\varPhi }^{+}\right\rangle$ obtained from full-basis QST, for all pairs of nuclear spins. The errors are obtained from Monte Carlo bootstrap resampling and represent 1*σ* from the mean. The graphs show nuclear spins 1 and 2 (**b**), nuclear spins 1 and 3 (**c**) and nuclear spins 2 and 3 (**d**) (see schematics). In the reconstructed density matrices, the filled bars represent experimental amplitudes, while the open bars indicate the amplitudes for an ideal $\left\vert {\varPhi }^{+}\right\rangle$ state. **e**–**g**, Two-qubit RB (orange) and two-qubit interleaved RB (purple), for all pairs of nuclear spins, corresponding to the same pairs of spins studied in **b** (**e**), **c** (**f**) and **d** (**g**). The physical CZ gate fidelites (${F}_{{\rm{CZ}}}^{12}$, ${F}_{{\rm{CZ}}}^{13}$ and ${F}_{{\rm{CZ}}}^{23}$) are calculated from the non-interleaved reference Clifford fidelities (${F}_{{\rm{C,ref}}}^{12}$, ${F}_{{\rm{C,ref}}}^{13}$ and ${F}_{{\rm{C,ref}}}^{23}$) and the interleaved Clifford fidelities (obtained from the fits to the purple data points). The errors are obtained from the least-square fits at a level of 1 s.d. The physical CZ gate fidelities for all pairs of nuclear spins are above the fault-tolerant threshold. Avg., average.

We use this gate to create all of the four Bell states, $\left\vert {\varPhi }^{\pm }\right\rangle =(\left\vert \Downarrow \Downarrow \right\rangle \pm \left\vert \Uparrow \Uparrow \right\rangle )/\sqrt{2}$, $\left\vert {\varPsi }^{\pm }\right\rangle =(\left\vert \Downarrow \Uparrow \right\rangle \pm \left\vert \Uparrow \Downarrow \right\rangle )/\sqrt{2}$, for each pair of nuclear spins (see Fig. 2a for the circuit diagram for nuclear spins 1 and 2). At the end of each measurement, we perform full-basis quantum state tomography (QST) to reconstruct the density matrix, *ρ*^*ij*^, and obtain the corresponding Bell state fidelity from ${F}_{{\rm{BS}}}^{\,ij}=\left\langle \psi | {\rho }^{ij}| \psi \right\rangle$, where *ψ* is the target Bell state and *i*, *j* = 1, 2 or 3 label the nuclear spins (Supplementary Section VI). Figure 2b–d shows the reconstructed density matrices for $\left\vert {\varPhi }^{+}\right\rangle$ for each pair of nuclear spins, with the Bell state fidelities listed in the tables above with state preparation and measurement (SPAM) errors included. The density matrices for $\left\vert {\varPhi }^{-}\right\rangle$ and $\left\vert {\Psi }^{\pm }\right\rangle$ are shown in Supplementary Section VII. We achieve average Bell state fidelities of ${F}_{{\rm{BS}}}^{12}=(97.5\pm 0.3) \%$, ${F}_{{\rm{BS}}}^{13}=(97.7\pm 0.4) \%$ and ${F}_{{\rm{BS}}}^{23}=(96.8\pm 0.4) \%$ for the three pairs of nuclear spins, among the highest fidelities that have been reported for spin qubits in Si (refs. ^13,31^).

Bell state fidelities are affected by SPAM errors, single- and two-qubit gate errors, and errors that occur when qubits idle. To independently quantify the fidelity of the CZ gate, we perform two-qubit RB and two-qubit interleaved RB with the CZ gate as the interleaved gate (Supplementary Section VIII). As shown in Fig. 2e–g, we find CZ gate fidelities of ${F}_{{\rm{CZ}}}^{12}=(99.65\pm 0.35) \%$, ${F}_{{\rm{CZ}}}^{13}=(99.49\pm 0.39) \%$ and ${F}_{{\rm{CZ}}}^{23}=(99.32\pm 0.22) \%$ for the three pairs of nuclear spins. In Supplementary Section IX, we show that the residual infidelities of the CZ gates can be explained by quasistatic variation in the electron energy splitting, which has been drastically reduced in this device by using isotopically pure silicon-28 material grown at low temperature using molecular beam epitaxy^28^. Two-qubit gate fidelities above the fault-tolerant threshold remain scarce in Si spin qubits and have only recently been reported^8,11–14,23^.

**Fig. 3:**
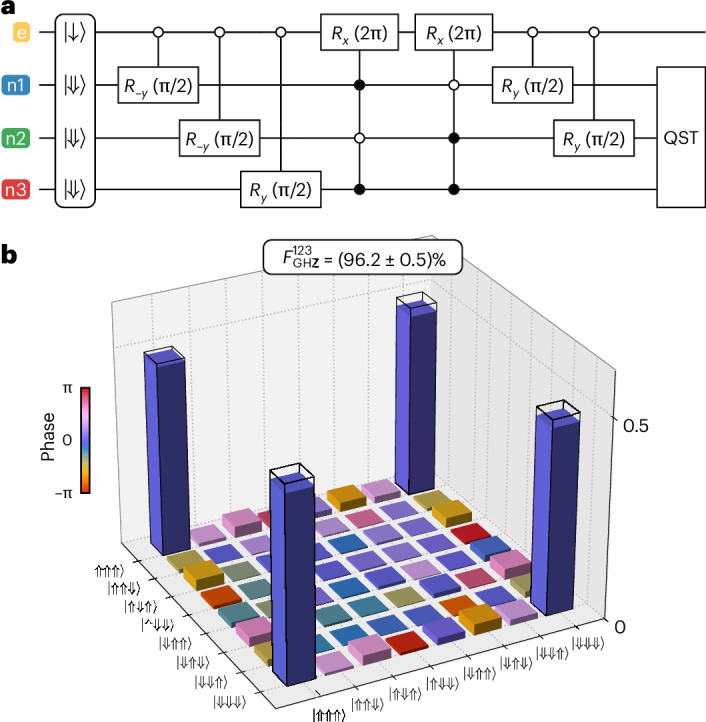
Three-qubit GHZ state tomography. **a**, The circuit diagram used to construct the GHZ state. **b**, The reconstructed density matrix of the GHZ state and corresponding state fidelity (${F}_{{\rm{GHZ}}}^{123}=(96.2\pm 0.5) \%$) as obtained from full-basis QST. The error is obtained from Monte Carlo bootstrap resampling and represents 1 s.d. from the mean. The filled bars represent experimental amplitudes, while the open bars represent amplitudes of an ideal GHZ state.

As a final demonstration of our ability to create entangled states, we entangle all three nuclear spins to create a GHZ state using the circuit in Fig. 3a. The reconstructed density matrix obtained from full-basis QST is shown in Fig. 3b. We achieve a fidelity of ${F}_{{\rm{GHZ}}}^{123}=(96.2\pm 0.5) \%$ (including SPAM errors), among the highest GHZ state fidelities reported for semiconductor spin qubits so far (see Supplementary Table 1 in Supplementary Section I).

## Grover’s search algorithm

**Fig. 4:**
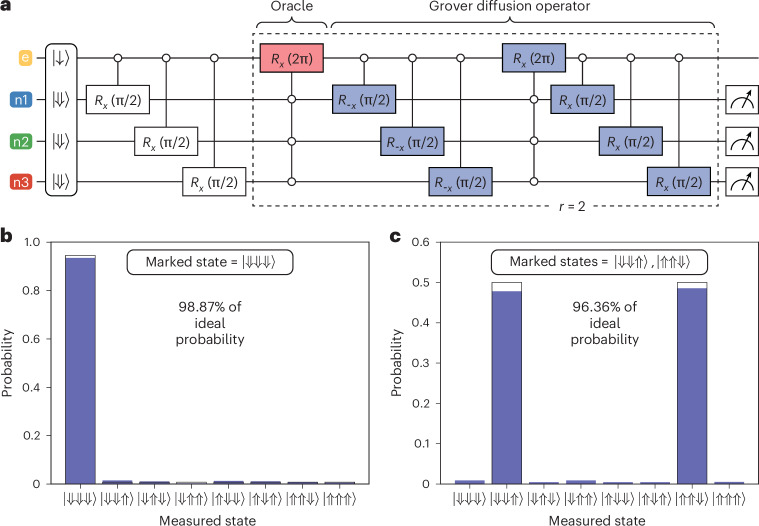
Three-qubit Grover’s algorithm. **a**, The circuit diagram implementing Grover’s algorithm on three nuclear spins. The oracle is highlighted in red (here marking the $\left\vert \Downarrow \Downarrow \Downarrow \right\rangle$ state), and the Grover diffusion operator is highlighted in blue. **b**, The measurement result when using $\left\vert \Downarrow \Downarrow \Downarrow \right\rangle$ as the marked state and performing *r* = 2 Grover iterations. **c**, The measurement result when marking the states $\left\vert \Downarrow \Downarrow \Uparrow \right\rangle$ and $\left\vert \Uparrow \Uparrow \Downarrow \right\rangle$, and performing *r* = 1 Grover iteration. For both **b** and **c**, the filled bars represent experimental probabilities, while the open bars represent the ideal performance of Grover’s algorithm if implemented on a quantum device with no errors.

Finally, we benchmark our four-qubit quantum processor by executing the well-known Grover’s search algorithm^32^, using the corresponding circuit shown in Fig. 4a. In general, this algorithm finds a specific bit string, *x*_*m*_, in the domain *x* of a function *f*, where *f* is defined such that it gives *f*(*x*_*m*_) = 1 and *f*(*x*_*i*_) = 0 for all other *x*_*i*_ ≠ *x*_*m*_. In our case, the domain consists of the eight binary values {000, 001, …, 111}, which correspond to the eight possible nuclear spin states $\{\left\vert \Downarrow \Downarrow \Downarrow \right\rangle ,\left\vert \Downarrow \Downarrow \Uparrow \right\rangle ,\ldots ,\left\vert \Uparrow \Uparrow \Uparrow \right\rangle \}$. Grover’s algorithm works by accessing *f* with a unitary operator (called oracle), ${U}_{{x}_{m}}$, which performs the action ${U}_{{x}_{m}}\left\vert x\right\rangle ={(-1)}^{\,f(x)}\left\vert x\right\rangle$. That is, the searched-for state (*x*_*m*_) is marked with a negative phase, while all other states are left unchanged. Taking advantage of the all-to-all connectivity in our processor, this oracle operation can be performed on the three nuclear spins by applying a single 2π rotation of the electron spin at the ESR frequency corresponding to *x*_*m*_ (highlighted in red in the circuit diagram in Fig. 4a). To find the marked state with high probability, the Grover iteration consisting of the oracle and the Grover diffusion operator (highlighted in blue in the circuit diagram and implementing the unitary $2{I}^{\otimes 3}-{(\left\vert +\right\rangle \left\langle +\right\vert )}^{\otimes 3}$) must be applied multiple times. Note that the Grover diffusion operator also benefits from all-to-all connectivity, requiring only a single entangling gate. For *n* = 3 qubits, the optimal number of repetitions is *r* = 2, which can be found using $r={{\rm{argmax}}}_{r}{\sin }^{2}\left[(2r+1)\arcsin \left({2}^{-n}\right)\right]$, where argmax takes the earliest local maximum^33^.

In Fig. 4b, we demonstrate the final measurement outcome of Grover’s algorithm executed on the three nuclear spins when using $\left\vert \Downarrow \Downarrow \Downarrow \right\rangle$ as the marked state. The algorithm finds this state with a probability of 93.46%, which corresponds to 98.87% of the ideal probability (94.53%) of finding the marked state with *r* = 2 Grover iterations. We also run the algorithm for all other marked states (Supplementary Section X) and find an average probability of (89.40 ± 2.49)% of finding the marked state, which corresponds to (94.57 ± 2.63)% of the ideal value. The uncertainties represent 1 s.d. from the mean taken over all marked states. This average fidelity is in good agreement with the predicted fidelity based on considering all the errors during qubit operations characterized in this work, as shown in Supplementary Section XI. In addition, we show that the algorithm can be run with two marked states using *r* = 1, which we would expect to achieve a success probability of 100%. Here, we achieve a probability of 96.36% to find the two marked states (Fig. 4c). These results represent one of the most successful implementations of Grover’s algorithm among any qubit platform so far (Supplementary Section XII).

## Conclusions

We have shown full coherent control in a four-qubit silicon processor consisting of three nuclear spins and one electron spin. The all-to-all qubit connectivity along with the long coherence times of the spin qubits allowed us to obtain control fidelities above the fault-tolerant threshold and to successfully execute a three-qubit Grover’s search algorithm with high accuracy.

Although in this work we used the electron spin to provide connectivity and to efficiently implement multi-qubit gates, it can also be used to couple neighbouring nuclear spin registers via the electron–electron exchange interaction. Exciting progress has been made in this direction^34–36^, and we anticipate the advent of quantum processors consisting of multiple connected registers in the near future. At the same time, as the placement precision of the STM approaches the atomic limit^37–39^, we anticipate future generations of devices with precisely engineered hyperfine interactions and tunnel couplings.

## Methods

### Fabrication

The device was fabricated using hydrogen STM lithography on a silicon chip with a 45 nm layer of isotopically purified silicon-28 (~200 ppm of residual Si-29 atoms). This epitaxial buffer layer decouples the device from the nuclear spin bath of the natural silicon substrate. After patterning the device, the sample is dosed with phosphine gas, followed by an incorporation anneal at 350 °C for 60 s to incorporate P atoms into the silicon crystal lattice. A 45 nm epitaxial layer of Si-28 is then grown at 250 °C and at a rate of 0.15 nm min^−1^ to ensure high-quality epitaxy. The buffer layer separates the qubits from any charged defects on the silicon surface and, together with the encapsulation layer, provides a monolithic qubit environment with minimal levels of spin and charge noise^40^. An STM image of the device is shown in fig. 1c,d in ref. ^28^, where the same device as studied in this work was also studied.

### Statistics and reproducibility

No statistical method was used to predetermine sample size. No data were excluded from the analyses. The experiments were not randomized. The investigators were not blinded to allocation during experiments and outcome assessment.

## Online content

Any methods, additional references, Nature Portfolio reporting summaries, source data, extended data, supplementary information, acknowledgements, peer review information; details of author contributions and competing interests; and statements of data and code availability are available at 10.1038/s41565-024-01853-5.

## Supplementary information

Supplementary Information Supplementary Sections I–XII, including Supplementary Figs. 1–6 and Supplementary Tables 1–4.

## Data Availability

The raw data used in this Article are available via Zenodo at 10.5281/zenodo.14214375 (ref. ^41^).

## Code availability

The code used to analyse the data and produce the figures in this Article is available via Zenodo at 10.5281/zenodo.14214375 (ref. ^41^).
