# Supplementary material for: Grover’s algorithm in a four-qubit silicon processor above the fault-tolerant threshold
Source: Nat Nanotechnol. 2025 Feb 20;20(4):472–7. doi: 10.1038/s41565-024-01853-5 (PMC12014505; doi:10.1038/s41565-024-01853-5)
Supplement: Supplementary file 1 — Supplementary Sections I–XII, including Supplementary Figs. 1–6 and Supplementary Tables 1–4. [file 41565_2024_1853_MOESM1_ESM.pdf]

nature nanotechnology

Supplementary information

https://doi.org/10.1038/s41565-024-01853-5

Grover's algorithm in a four-qubit silicon processor above the fault-tolerant threshold

In the format provided by the authors and unedited

CONTENTS

I. State-of-the-art semiconductor spin qubit processors 2
II. Nuclear spin non-demolition readout 2
III. Nuclear spin initialization 5
IV. Dephasing time of the qubits 5
V. Single-qubit Randomized Benchmarking 6
VI. Quantum State Tomography 7
VII. Density matrices for all Bell states 7
VIII. Two-qubit Randomized Benchmarking 8
IX. Nuclear-Nuclear Two-Qubit Gate Errors 9
X. Additional data for Grover's algorithm 12
XI. Error budget for Grover's Algorithm 12
XII. State-of-the-art implementations of Grover's algorithm 13
References 15

I. STATE-OF-THE-ART SEMICONDUCTOR SPIN QUBIT PROCESSORS

TABLE SI. **Comparison of state-of-the-art semiconductor spin qubit quantum processors using gate-defined quantum dots in Si/SiGe and in Ge/SiGe and using multi-nuclear Si:P spin registers.** We only include processors that have demonstrated an algorithm or QEC (for two-qubit processors we only include those with single and two-qubit gate fidelities above 99%). Furthermore, we include the processor with the largest number of coherently controlled qubits using gate-defined quantum dots in Si and using Si:P spin registers. “SPAM” stands for “state preparation and measurement”, “VQE” for “variational quantum eigensolver”, and “DJ” for “Deutsch-Jozsa”.

Reference	Xue [1]	Noiri [2]	Takeda [3]	Philips [4]	Hendrickx [5] Van Riggelen [6]	Madzik [7]	This work
Year	2022	2022	2022	2022	2021/2022	2022	2024
Platform	Si/SiGe	Si/SiGe	Si/SiGe	Si/SiGe	Ge/SiGe	Si:P	Si:P
Qubits	2 (electrons)	2 (electrons)	3 (electrons)	6 (electrons)	4 (holes)	3 (n-n-e)	4 (n-n-n-e)
SPAM fidelity (%)	-	74.25^{a}	-	-	-	98.95^{a} (n)	99.42 to 99.57 (n)
Rabi visibility (%)	-	-	70 to 85^{b}	93.5 to 98^{c}	60 to 75^{b}	-	92 to 99 (n)
Single-qubit gate fidelity (%)	99.71 to 99.74	99.84 to 99.84	99.68 to 99.77	99.77 to 99.96	99.40 to 99.88	99.46 to 99.91 (n)	99.95 to 99.98 (n)
Two-qubit gate fidelity (%)	99.65	99.51	-	-	-	99.37 (n-n)	99.32 to 99.65 (n-n)
Bell state fidelity (%)	98.1^{d} (w/o SPAM)	96.5 (w/o SPAM)	-	78.0 to 91.3	-	93.4 (n-n)	96.8 to 97.7 (n-n)
Three-qubit GHZ state fidelity (%)	N/A	N/A	86.6 (w/o SPAM)	52.7 to 67.2	-	92.5^{e} (n-n-e)	96.2 (n-n-n)
Demonstration of algorithm or QEC	Two-qubit VQE algorithm	Two-qubit DJ and Grover’s algorithm	Three-qubit phase-flip QEC code	-	Three-qubit phase-flip QEC code	-	Three-qubit Grover’s algorithm

^{a} Average two-qubit SPAM fidelity as stated in references [2, 7].
^{b} Rabi visibility estimated from Extended Data Fig. 2b-d in [3] and Fig. 1f in [5].
^{c} Values for operating individual qubits; when initializing other qubits in the device the Rabi visibilities decrease as stated in reference [4].
^{d} From simulation (rather than measurement) as stated in reference [1].
^{e} From return probability (rather than quantum state tomography) as stated in reference [7].

II. NUCLEAR SPIN NON-DEMOLITION READOUT

The nuclear spins are read out via the electron spin (a detailed description of electron spin readout is provided in [8]). This nuclear spin measurement is quantum non-demolition, i.e. the nuclear spin remains in the projected measured state after the measurement operation. For every nuclear spin we perform N readout shots, each shot consisting of the following operations (see Fig. S1a): initialization of the electron spin into the $|\downarrow\rangle$ state, adiabatic inversion of the electron spin conditional on the nuclear spin being in the $|\Downarrow\rangle$ state, electron spin readout, initialization of the electron spin into the $|\downarrow\rangle$ state, adiabatic inversion of the electron spin conditional on the nuclear spin being in the $|\Uparrow\rangle$ state, and finally electron spin readout. From this sequence of measurements, we obtain the fraction of shots detecting nuclear spin down ($f_{\Downarrow} = N_{\Downarrow}/N$) and nuclear spin up ($f_{\Uparrow} = N_{\Uparrow}/N$), where $N_{\Downarrow/\Uparrow}$ is the number of electron spin up events detected for the specific nuclear spin state. If the value of $\Delta f = f_{\Uparrow} - f_{\Downarrow}$ is positive we assign a nuclear state $|\Uparrow\rangle$, and for $\Delta f \leq 0$ we assign a nuclear state $|\Downarrow\rangle$. For repeated measurements (each consisting of N readout shots), we can form a histogram of the observed values of Δf. Examples of this are shown in Fig. S1c-e, where we observe two well separated Gaussian peaks corresponding to the nuclear spin state $|\Uparrow\rangle$ and $|\Downarrow\rangle$. To maximize the readout fidelity for every nuclear spin for the tomography and Grover’s algorithm data obtained in this work, we optimize the number of readout shots and postselect the observed nuclear readouts, keeping only those readouts where

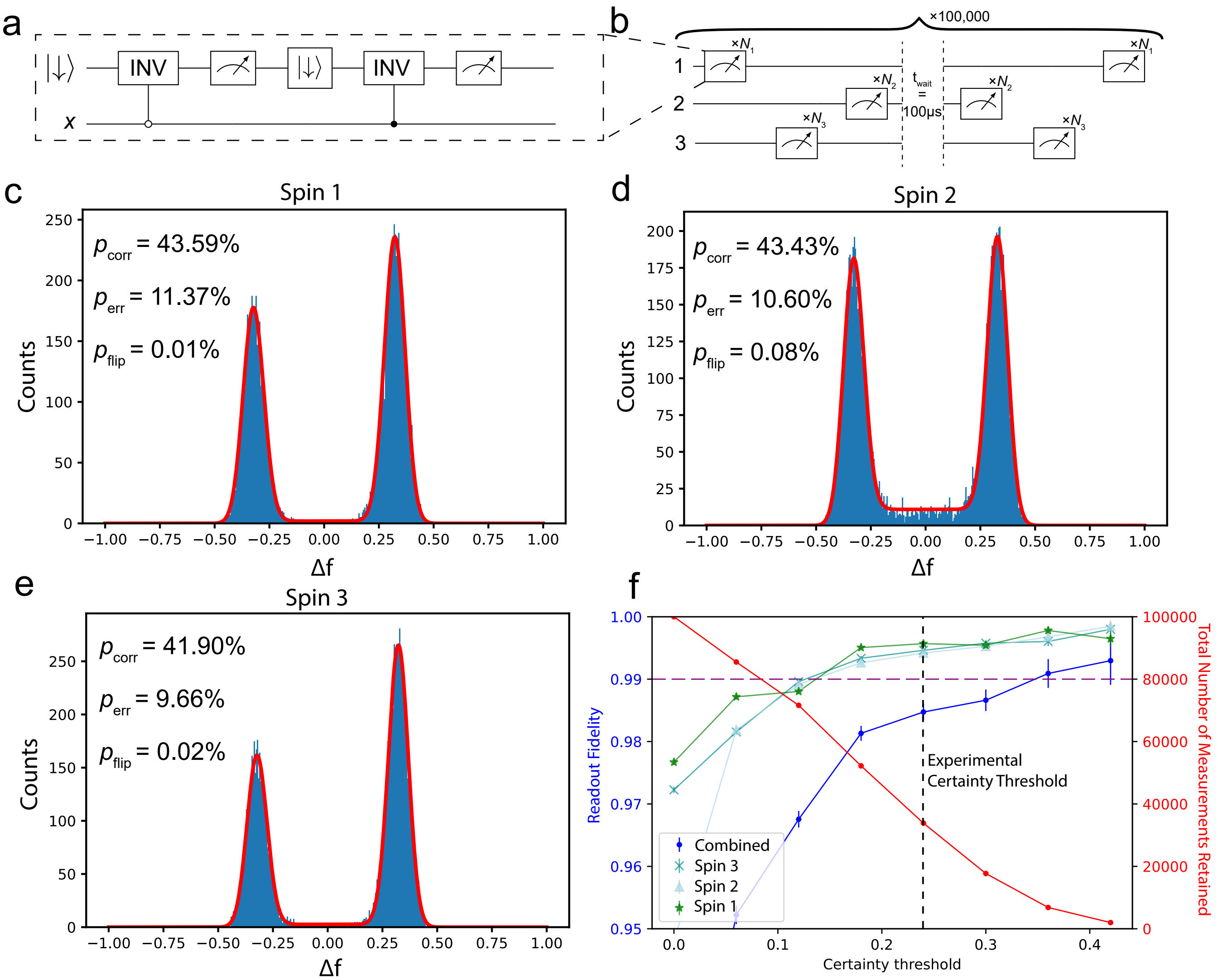

FIG. S1. **Optimization of nuclear spin non-demolition readout.** **a** Circuit used to perform a single shot of readout of one of the nuclear spins, with $x = 1, 2, 3$ being the label for the nuclear spin that is read out. **b** Circuit used to measure the readout fidelity of the nuclear spins. This circuit is repeated 100,000 times to obtain the readout fidelities. **c-e** Histograms of Δf for 10,000 repeated readout measurements for all three individual nuclear spins. Each measurement involves taking $N = 200$ shots of the corresponding nuclear spin. Included are fits from a Markov chain model (red lines) to extract $p_{\rm corr}$, $p_{\rm err}$, and $p_{\rm flip}$. **f** Individual and combined nuclear spin readout fidelities (left axis) and number of measurements retained (right axis) as a function of the certainty threshold for (N_1,N_2,N_3)=(24,18,24). Note that different certainty thresholds are used for each nuclear spin, with the threshold displayed on the x-axis being scaled by $(\frac{1}{2}, 1, \frac{2}{3})$ for spins 1, 2 and 3 respectively. The error bars for (f) are calculated according to $\sigma_f = \sqrt{1 + 4f(1-f)n}\ /2(n+1)$, representing the range of true fidelities for which the observed fidelity lies within 1σ of the relevant binomial distribution, as defined in this section.

$|\Delta f|$ lies above a defined "certainty threshold" ($f_{\rm th}$). This certainty threshold is designed to remove measurements where the nuclear spin flipped during the nuclear non-demolition readout.

To find the optimal number of readout shots for each nuclear spin (N_1,N_2,N_3), we model the nuclear spin readout histograms using a Markov chain model. This model contains three parameters, the probability of correctly observing an electron blip when driving the peaks corresponding to the nuclear spin's true state ($p_{\rm corr}$), the probability of incorrectly observing an electron blip when driving the peaks corresponding to the opposite of the nuclear spin's true state ($p_{\rm err}$), and the probability of the nuclear spin flipping during the shot ($p_{\rm flip}$). We find these parameters by fitting the model to experimental readout histograms. The histograms along with the fits and the extracted parameters are shown in Fig. S1c-e. The non-zero counts between the peaks correspond to nuclear spin flips during the nuclear spin

readout. After obtaining p_{corr}, p_{err} and p_{flip}, we vary the number of readout shots for each nuclear spin from 1-50, and calculate the individual nuclear spin readout fidelities (F_1,F_2,F_3) from the modelled readout histograms. To obtain the optimal number of shots that maximizes the combined nuclear spin readout fidelity (F_N=$F_1F_2F_3$), we first note that due to the extremely low probability of nuclear spin 3 flipping during a shot ($p_{\text{flip},3} \approx 0.01$ %), the readout fidelity for nuclear spin 3 saturates after $\sim$ 19 shots. We therefore set $N_3 = 19$. We find that the highest modelled fidelity $F_N = 97.90\%$ occurs at (N_1,N_2,N_3)=(19,13,19), and using these values as a guide, we find experimentally that the highest fidelity occurs in a similar parameter regime: (N_1,N_2,N_3)=(24,18,24). We use these values for all measurements that are sensitive to the readout fidelity (QST of the Bell states and of the GHZ state, as well as Grover's algorithm). When measuring all spins at the end of a circuit, the spins are measured in the order (spin 2, spin 3, spin 1), so that spins with higher error are measured first before large errors can accumulate and therefore reduce measurement fidelity. Similarly, when measuring all spins at the start of a circuit (for example to verify that initialisation was successful), spins are measured in the order (spin 1, spin 3, spin 2) to ensure the verification of spins with highest error occurs as close to the start of the circuit as possible, minimising the time for large errors to occur between verification and the circuit.

To measure the readout fidelity for a given N_1, N_2 and N_3, we perform an experiment where we read out all three nuclear spins, wait for 100 μs, and then read out all three nuclear spins again (see Fig. S1b). This sequence is repeated 100,000 times. We define the readout fidelity as the proportion of repetitions where the first and second readout yield the same nuclear spin configuration in relation to the total number of repetitions. In Fig. S1f we show the individual and combined nuclear spin readout fidelities as a function of the relative certainty threshold for (N_1,N_2,N_3)=(24,18,24). The error bars used in Fig. S1f are calculated so as to show the range of possible true fidelities which would provide the observed fidelity within 1σ, assuming the measurement results are binomially distributed. Explicitly, they are calculated according to the formula:

$$\sigma_f = \frac{\sqrt{1 + 4f(1-f)n}}{2(n+1)} \tag{1}$$

where σ_f is the calculated uncertainty, f is the experimentally sampled fidelity, and n is the total number of measurements remaining after certainty thresholding. In Fig. S1f, certainty thresholds for individual spins are scaled by factors of $(\frac{1}{2}, 1, \frac{2}{3})$ respectively, postselecting spin 2 more strictly than the others because of its higher error. Throughout this work we use $f_{\text{th}} = 0.24$ for spin 2 (and relative scaled values for other nuclei), retaining $\sim 33\%$ of readout measurements with readout fidelities of 99.46%, 99.42%, and 99.57%.

III. NUCLEAR SPIN INITIALIZATION

To initialize the nuclear spins into the desired state (e.g. $|\Downarrow\Downarrow\Downarrow\rangle$), we use a process called electron state transfer (EST) consisting of a sequence of ESR and NMR pulses, previously demonstrated in nitrogen vacancies in diamond [9]. EST is performed at the beginning of each circuit and therefore repeated for every circuit repetition.

EST initializes the nuclear spins sequentially. In order to initialize the first nuclear spin, we use the following sequence: starting from an unknown nuclear spin state, we first initialize the electron spin into the down state. Then we apply the four adiabatic ESR pulses that correspond to flipping the electron spin conditional on the first nuclear spin being in the unwanted state. This is followed by an NMR π-rotation conditional on the electron spin being in the up state. The combination of electron spin down initialization, the four ESR pulses and the NMR pulse flips the first nuclear spin if it is in the unwanted state and leaves it untouched otherwise (since then the electron spin is not flipped to the up state by the ESR pulses). We repeat this sequence for the other two nuclear spins, leading to a fully initialized nuclear spin register. To verify that the nuclear spins are indeed in the correct state, we perform a non-demolition readout of the nuclear spins.

Since a verification readout is performed to ensure that the nuclear spins are initialised correctly, after postselecting on the verification readout the nuclear spin initialisation fidelities are the same as the nuclear spin readout fidelities. As discussed in Supplementary section II, this means that postselected nuclear spin initialisation fidelities are above 99% for all nuclear spins.

IV. DEPHASING TIME OF THE QUBITS

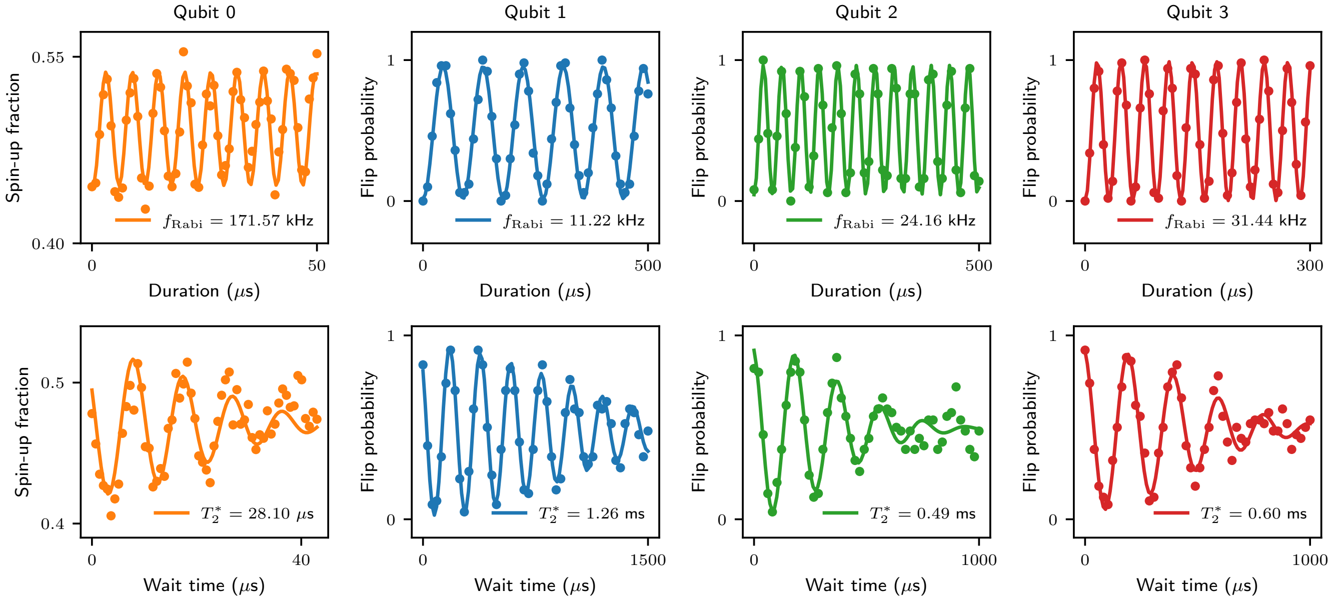

FIG. S2. **Rabi oscillations (top row) and Ramsey measurement (bottom row) for each qubit.** The Rabi frequencies and T_2^* times are indicated for each qubit. For the electron spin qubit, the nuclear spins were initialized into the $|\Downarrow\Downarrow\Downarrow\rangle$ state; for the nuclear spin qubits, the electron spin was initialized into the $|\downarrow\rangle$ state.

The dephasing times of the four qubits that we quote in the main text are extracted from Ramsey experiments. For the electron spin qubit, we first initialize the nuclear spins into the $|\Downarrow\Downarrow\Downarrow\rangle$ state using EST, then we initialize the electron spin into the $|\downarrow\rangle$ state, followed by an $R_x(\pi/2)$ rotation, an identity gate with varied wait time, another $R_x(\pi/2)$ rotation, and finally readout of the electron spin (all gates are applied conditional on the nuclear spins being in the $|\Downarrow\Downarrow\Downarrow\rangle$ state). For the nuclear spin qubits, we start by performing a non-demolition readout of the nuclear spins to determine the initial nuclear spin state, followed by the initialization of the electron spin into the $|\downarrow\rangle$ state. We

then perform the Ramsey sequence with NMR gates conditional on the electron spin being in the $|\downarrow\rangle$ state, and a final non-demolition readout.

We fit the Ramsey measurements according to

$$A \cdot \sin(\omega t + \phi) \exp\left(-(t/T_2^*)^2\right) + B, \tag{2}$$

where t is the wait time, and A, ω, ϕ and B are fitting parameters. Rabi measurements are fit according to

$$A \cdot \sin(\omega t + \phi) + B, \tag{3}$$

the same form as the Ramsey measurements, but without the decay. Figure S2 shows the Ramsey measurements and the fits for the four qubits (bottom row) along with Rabi oscillations for each qubit (top row).

We also measured the Rabi frequency and the dephasing time for the electron spin with the nuclear spins initialized into all other configurations, and the results are summarized in Table SII. Note that due to frequency-dependent attenuation in the cables within the dilution refrigerator, the drive amplitudes for each ESR peak have been adjusted to have all ESR rabi frequencies in the proximity of 170 kHz, and that these settings are used throughout the rest of this work. In addition, the Rabi dephasing time was measured for all qubits, as summarised in Table SII.

Qubit	f_{Rabi} (kHz)	T_2^* (μs)	T_2^{Rabi} (ms)
e ($\|\Downarrow\Downarrow\Downarrow\rangle$)	171.57	28.10	0.191
e ($\|\Downarrow\Downarrow\Uparrow\rangle$)	170.67	31.43	-
e ($\|\Downarrow\Uparrow\Downarrow\rangle$)	172.27	33.60	-
e ($\|\Downarrow\Uparrow\Uparrow\rangle$)	172.01	30.79	-
e ($\|\Uparrow\Downarrow\Downarrow\rangle$)	168.63	26.71	-
e ($\|\Uparrow\Downarrow\Uparrow\rangle$)	171.04	38.26	-
e ($\|\Uparrow\Uparrow\Downarrow\rangle$)	170.64	37.75	-
e ($\|\Uparrow\Uparrow\Uparrow\rangle$)	171.29	26.73	-
n1	11.22	1260	71.75
n2	24.16	490	3.50
n3	31.44	600	2.21

TABLE SII. **Rabi frequency, dephasing time and Rabi dephasing time for the electron and nuclear spin qubits.** For the electron spin, Rabi and dephasing times are measured with the nuclear spins initialized into the different configurations as depicted. Note that the Rabi dephasing time for the electron spin was only measured for the nuclear state $|\Downarrow\Downarrow\Downarrow\rangle$. The T_2^* times for the nuclear spin qubits are given to the nearest 10 μs.

V. SINGLE-QUBIT RANDOMIZED BENCHMARKING

To perform single-qubit randomized benchmarking for the electron spin qubit or the nuclear spin qubits, we first initialize all spins. Afterwards we apply a specific number (N) of randomly chosen Clifford gates (each Clifford gate consists of 1.875 physical gates on average), followed by a recovery gate to spin up in the first circuit repetition and spin down in the second repetition, and a readout of the electron or nuclear spin. We then repeat this sequence for varying N. Finally, the whole experiment is repeated a number of times (20 for the electron spin and 15 for the nuclear spins), where in every repetition a new randomly chosen set of Clifford gates is applied for every N.

From this experiment we obtain two decay curves (after averaging over all random variations per N), one with recovery to spin up (P^{u}) and one with recovery to spin down (P^{d}). We combine the two curves into a single one according to

$$P = (P^{\mathrm{u}} + (1 - P^{\mathrm{d}}))/2, \tag{4}$$

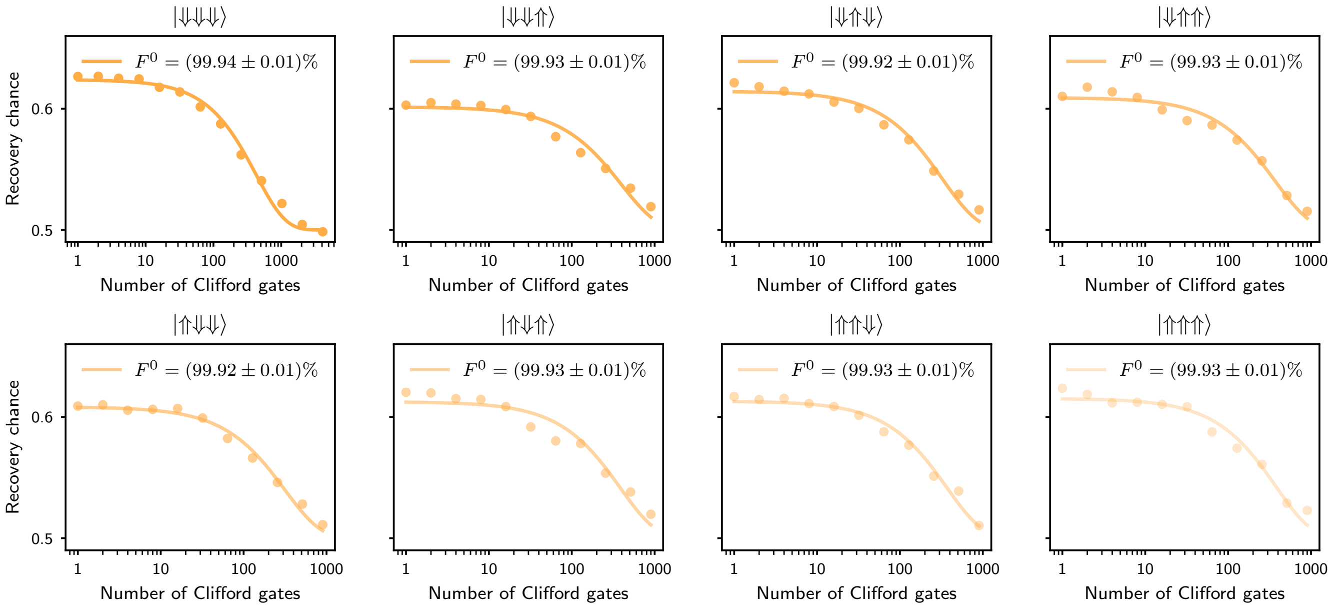

FIG. S3. **Electron spin randomized benchmarking with the nuclear spins initialized into all possible configurations.** The errors for the fidelities are obtained from the fits.

which we fit with $af^N + 0.5$, and obtain the Clifford gate fidelity from $F_C^i = 1 - (1 - f)/2$, where $i = 0, 1, 2, 3$ labels the qubit [10]. We then calculate the physical gate fidelity from $F^i = 1 - (1 - F_C^i)/1.875$.

In Fig. 1d of the main text we show RB for the electron spin with the nuclear spins initialized into the $|{\Downarrow\Downarrow\Downarrow}\rangle$ state. We also performed RB with the nuclear spins initialized into all other states (using EST as described in Supplementary section III), where we find Clifford gate fidelities above 99% for all nuclear spin configurations, as shown in Fig. S3.

VI. QUANTUM STATE TOMOGRAPHY

To perform quantum state tomography (QST) on two or three nuclear spin qubits, we measure the qubits in all possible two- or three-qubit Pauli-bases respectively, which we achieve by applying single-qubit NMR rotations prior to the nuclear non-demolition readout. To measure a nuclear spin in the x-basis we perform a nuclear $R_{-y}(\pi/2)$ rotation conditional on the electron spin being in the $|{\downarrow}\rangle$ state, to measure in the y-basis we apply a $R_x(\pi/2)$ rotation conditional on the electron spin being in the $|{\downarrow}\rangle$ state, and to measure in the z-basis we apply no rotation prior to the non-demolition readout. We apply the rotations in the order (qubit 3, qubit 2, qubit 1), to minimise qubit idle/dephasing time by performing slower rotations first, and perform non-demolition readout of the nuclear spins in the order (qubit 2, qubit 3, qubit 1) so that nuclear spins with higher error are measured first before significant errors can accumulate.

To obtain the density matrix from the tomography counts, we perform a constrained Gaussian linear least-squares fit to the count data. The errorbars are obtained from Monte Carlo bootstrap re-sampling and represent 1σ from the mean [11, 12].

VII. DENSITY MATRICES FOR ALL BELL STATES

In Fig. 2b-d of the main text we show the density matrix for one of the Bell states (Φ^+), for all pairs of nuclear spins. Figure S4 shows the density matrices for all Bell states (Φ^+, Ψ^+, Φ^-, Ψ^-), for all pairs of nuclear spins.

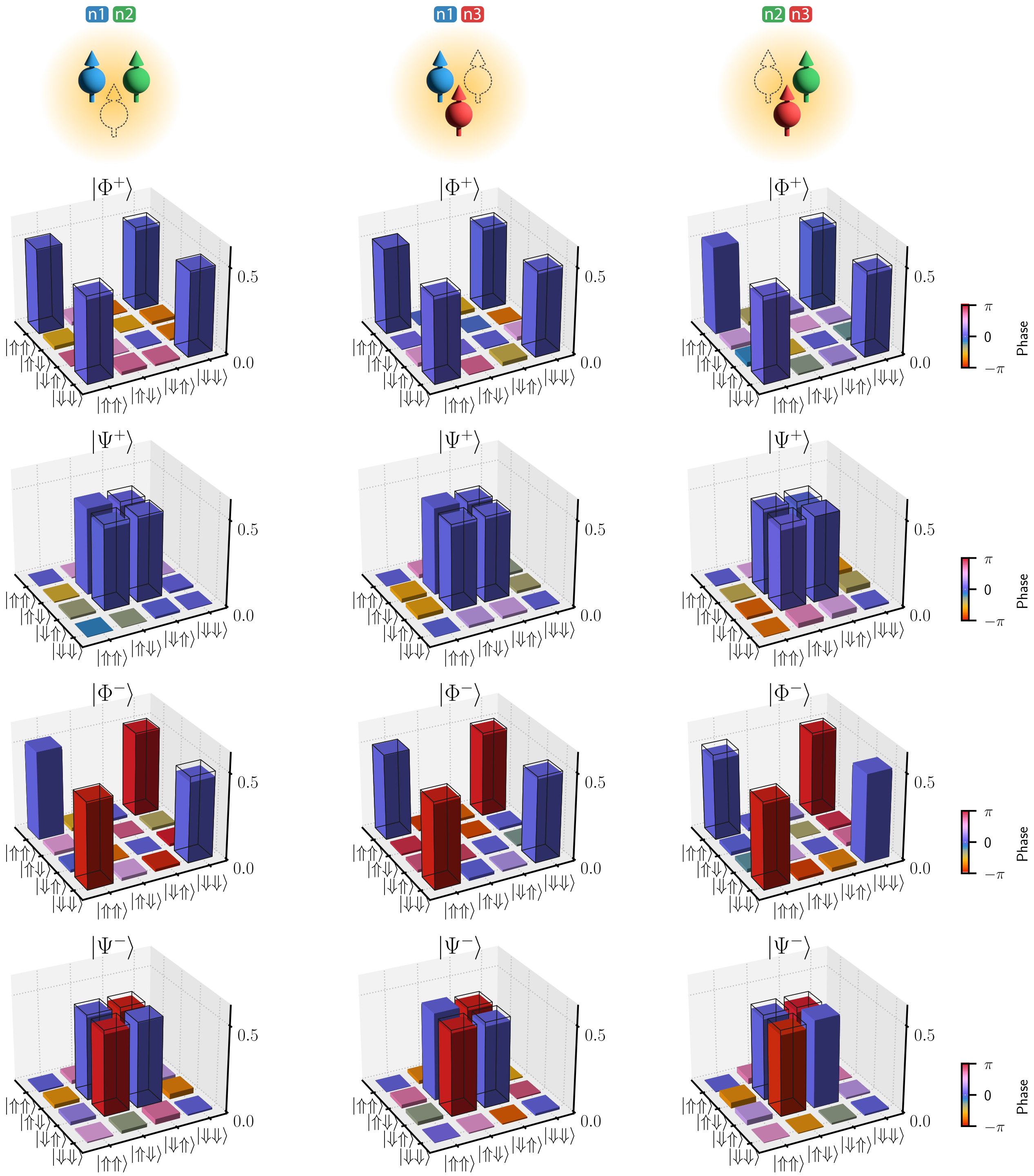

FIG. S4. **Density matrices for all Bell states, for all pairs of nuclear spins.** The left column corresponds to nuclear spin qubit 1 and 2, the middle column to qubit 1 and 3, and the right column to nuclear spin qubit 2 and 3.

VIII. TWO-QUBIT RANDOMIZED BENCHMARKING

To perform two-qubit RB we first initialize the nuclear spins into the $|{\Downarrow\Downarrow\Downarrow}\rangle$ state, followed by an initialization of the electron spin into the $|{\downarrow}\rangle$ state. Then we apply a fixed number (N) of Clifford gates (each Clifford gate consists on average of ~ 5.03 single-qubit $\pi/2$ rotation gates and 1.5 two-qubit CZ gates) and a recovery gate so that the total unitary is identity, returning the two nuclear spins to $|{\Downarrow\Downarrow}\rangle$. The decomposition of Cliffords into na-

tive operations is optimised to firstly minimise the number of CZ gates applied, and secondarily to avoid applying single-qubit gates to only one qubit for an extended period where possible (to avoid the other qubit dephasing). The sequence of N Clifford gates is followed by non-demolition readout of the nuclear spins. This sequence is repeated for varying N. Afterwards we repeat the experiment with new randomly chosen Clifford gates for every N. After 5 of these repetitions we perform 5 repetitions where we interleave a two-qubit CZ gate between every Clifford gate to perform interleaved two-qubit RB. Finally, we repeat the sequence of 5 non-interleaved and 5 interleaved repetitions 9 more times to arrive at a total of 50 non-interleaved and 50 interleaved final runs for each pair of nuclear spins.

From this measurement we obtain two RB decay curves (after averaging over all random variations per N), a non-interleaved (reference) curve and an interleaved curve. We fit both curves with $af^N + b$ and calculate the corresponding Clifford fidelity from $F^{ij}_{\mathrm{ref/int}} = 1 - 3(1 - f_{\mathrm{ref/int}})/4$, where $i, j \in [1, 2, 3]$ label the used nuclear spins. From the decay rate ratio ($d = f_{\mathrm{int}}/f_{\mathrm{ref}}$) we obtain the interleaved gate fidelity via $F^{ij}_{\mathrm{CZ}} = 1 - 3(1 - d)/4$ [13].

IX. NUCLEAR-NUCLEAR TWO-QUBIT GATE ERRORS

In this work, the multi-qubit gate between nuclear spins is achieved by performing a 2π rotation of the electron, conditional on a particular nuclear spin configuration, as discussed in the main text. The fidelity of this multi-qubit gate was characterised in Fig. 2 of the main text between each pair of nuclei, giving fidelities of 99.65 ± 0.35 %, 99.49 ± 0.39 % and 99.32 ± 0.22 %, giving an average value of 99.49 ± 0.20 %. Here, we explore how quasistatic variation of the electron energy splitting affects the performance of the 2π rotation, and show that the corresponding predicted error agrees with the observed experimental value. For ease of calculation, we work in angular frequency; ie. setting $\hbar = 1$.

When the electron is present, and in the absence of control pulses, the Hamiltonian of the multi-nuclear register is given by:

$$H = \left(\gamma_e S_z + \sum_{i=1}^{3} \gamma_n I_{i,z} \right) B_0 + \sum_{i=1}^{3} A_i \mathbf{S} \cdot \mathbf{I_i} \tag{5}$$

where γ_e (γ_n) is the electron (nuclear) gyromagnetic ratio, and A_i is the contact hyperfine coupling interaction strength for nuclear spin $i \in \{1, 2, 3\}$. To model the evolution of all qubits in this system, we work in the interaction picture. That is, we choose the unitary

$$U(t) = \exp\left(iHt\right) \tag{6}$$

and use it to transform the Hamiltonian and state as follows:

$$|\psi(t)\rangle \rightarrow U(t)\,|\psi(t)\rangle \tag{7}$$

$$H \rightarrow i\frac{\partial U(t)}{\partial t}U^{\dagger}(t) + U(t)HU^{\dagger}(t) = 0 \tag{8}$$

Working in the interaction picture ensures that when the qubits are idle, they will not pick up phase as the Hamiltonian is 0. We define the phase of our qubits in the interaction frame, so that any phase errors accumulated will be solely caused by errors in the magnetic control pulses.

To model the action of a control pulse, it is useful to temporarily tranform the system to a rotating frame which rotates at the frequency of the control pulse, making sure to return back to the original interaction frame at the end of the control pulse to establish the final phase of the qubits. As we are interested in the application of ESR control in this section, it is also valid to work in the secular approximation as $\gamma_e B_0 \sim 39$ GHz is much larger than other energy scales of the Hamiltonian (that is, we assume only the z-terms contribute in the Hamiltonian in Eq. 5). In addition, we apply the rotating wave approximation, as we are in the limit of weak driving. Under these approximations, for a two-level system with energy splitting E and a control pulse of frequency $E+\delta$ and drive strength ω, the Hamiltonian in a frame rotating at $E+\delta$ is given by:

$$H_d = -\frac{\delta}{2}\sigma_z + \frac{\omega}{2}\sigma_x \tag{9}$$

After applying this drive Hamiltonian for some time t, the unitary applied is given by:

$$U_d = \exp\left(-iHt\right) \tag{10}$$
$$= \sigma_I \cos\left(\sqrt{\delta^2+\omega^2}t/2\right) + i\frac{\delta\sigma_z - \omega\sigma_x}{\sqrt{\delta^2+\omega^2}} \sin\left(\sqrt{\delta^2+\omega^2}t/2\right) \tag{11}$$

If the control pulse is far off-resonance, ie. $\delta \gg \omega$, we have

$$U_d'(t) \approx \sigma_I \cos\left(\delta t/2\right) + i\sigma_z \sin\left(\delta t/2\right) \tag{12}$$
$$U_d'(t) \approx \exp\left(i\delta\sigma_z t/2\right) \tag{13}$$

Now, consider the application of a 2π rotation of the electron in the 4-qubit register considered in this work, conditional on the nuclear state $|\Downarrow\Downarrow\Downarrow\rangle$, with the electron starting $|\downarrow\rangle$. If the terms in Eq. 5 were known exactly, then such a control pulse (in the secular approximation) would be applied at a frequency of $\gamma_e B_0 - \left(\sum_i A_i\right)/2$, which is the energy splitting between $|\downarrow\Downarrow\Downarrow\Downarrow\rangle$ and $|\uparrow\Downarrow\Downarrow\Downarrow\rangle$ in the secular approximation. However, due to imprecise knowledge of the Hamiltonian, the applied frequency will actually be $\gamma_e B_0 - \left(\sum_i A_i\right)/2 + \delta$ for some small detuning δ (representing the difference between the experimentally calibrated energy splitting and the true energy splitting). To transform from the interaction picture to a frame rotating at this drive frequency, for the subspace $\{|\downarrow\Downarrow\Downarrow\Downarrow\rangle, |\uparrow\Downarrow\Downarrow\Downarrow\rangle\}$, one applies the unitary:

$$U_{\text{trans}}(t) = \exp\left(i\delta S_z t\right) \tag{14}$$

to account for the frequency difference δ between the interaction picture and our drive frequency. We assume that the pulse starts at $t = 0$, and finishes after the electron has returned to the $|\downarrow\rangle$ state at time $t = \tau \equiv 2\pi/\omega \approx 2\pi/\sqrt{\delta^2+\omega^2}$. Then the full unitary that is performed is given by:

$$U_{2\pi} = U_{\text{trans}}^\dagger(\tau)\, U_d(\tau)\, U_{\text{trans}}(0) \tag{15}$$
$$= U_{\text{trans}}^\dagger(\tau)\cdot(-I)\cdot(I) \tag{16}$$
$$U_{2\pi} = -\begin{bmatrix} e^{-i\delta\tau/2} & 0 \\ 0 & e^{i\delta\tau/2} \end{bmatrix} \tag{17}$$

which is the unitary applied to the subspace $\{|\downarrow\Downarrow\Downarrow\Downarrow\rangle, |\uparrow\Downarrow\Downarrow\Downarrow\rangle\}$. For all other pairs of states, the drive will be far from the energy splitting, so we instead use the drive Hamiltonian when far off-resonance (given in Eq. 13), which gives:

$$U_{2\pi}' = U_{\text{trans}}^\dagger(\tau)\, U_d'(\tau)\, U_{\text{trans}}(0) \tag{18}$$
$$\approx \exp\left(-i\delta S_z\tau\right)\cdot\exp\left(i\delta S_z\tau\right)\cdot(I) \tag{19}$$
$$U_{2\pi}' \approx I \tag{20}$$

Hence, for all pairs of states *except* the subspace $\{|\downarrow\Downarrow\Downarrow\Downarrow\rangle, |\uparrow\Downarrow\Downarrow\Downarrow\rangle\}$, the identity unitary is performed. Therefore, if we project into the electron-$|\downarrow\rangle$ subspace (limiting ourselves to a pair of nuclear spins, as we are concerned with the two-qubit gate fidelity), the unitary is given by:

$$U_{2\pi} = \begin{bmatrix} -e^{-i\delta\tau/2} & & & \\ & 1 & & \\ & & 1 & \\ & & & 1 \end{bmatrix} \tag{21}$$

The fidelity of this operation is given by [14]

$$F = \frac{1}{d^2}\left|\mathrm{tr}\left(U_{2\pi}^{\dagger}V_{2\pi}\right)\right|^2 \tag{22}$$

where d is the dimension of the Hilbert space, and $V_{2\pi}$ is the ideal intended unitary, ie. $U_{2\pi}|_{\delta=0}$. Substituting in Eq. 21, we get:

$$F = \frac{1}{16}\left|3 + e^{i\delta\tau/2}\right|^2 \tag{23}$$

$$= \frac{[3+\cos(\delta\tau/2)]^2 + \sin^2(\delta\tau/2)}{16} \tag{24}$$

$$\approx \frac{\left[4 - \frac{\delta^2\tau^2}{8}\right]^2 + (\delta\tau/2)^2}{16} + O(\delta^4\tau^4) \tag{25}$$

$$\approx \frac{16 - \delta^2\tau^2 + (\delta\tau/2)^2}{16} + O(\delta^4\tau^4) \tag{26}$$

$$F \approx 1 - \frac{3}{4}\left(\frac{\delta\tau}{4}\right)^2 + O(\delta^4\tau^4) \tag{27}$$

Hence, the average two-qubit error is given by

$$\epsilon = 1 - F \tag{28}$$

$$\epsilon \approx \frac{3}{4}\left(\frac{\delta\tau}{4}\right)^2 + O(\delta^4\tau^4) \tag{29}$$

In practice, the detuning δ will not be fixed, but instead will vary over time due to noise in the energy splitting of the electron. A significant component of this variation is quasistatic, meaning δ will vary repetition-to-repetition in an amount characterised by T_2^*. Specifically, the variance in δ (measured in angular frequency) caused by T_2^* is given by [15]:

$$\mathrm{Var}(\delta) = \frac{2}{(T_2^*)^2} \tag{30}$$

The expected error can be calculated as follows:

$$\mathrm{E}(\epsilon) = \int \epsilon(\delta)p(\delta)d\delta \tag{31}$$

$$\approx \int \frac{3}{4}\left(\frac{\delta\tau}{4}\right)^2 p(\delta)d\delta \tag{32}$$

$$\mathrm{E}(\epsilon) \approx \frac{3}{4}\left(\frac{\tau}{4}\right)^2 \mathrm{E}(\delta^2) \tag{33}$$

where E denotes expectation value. Assuming that the experiment is well-calibrated, meaning that $\mathrm{E}(\delta) = 0$, we can utilise the fact that $\mathrm{Var}(\delta) = \mathrm{E}(x^2) - [\mathrm{E}(x)]^2$ to show that $\mathrm{E}(\epsilon)$ is inversely proportional to the square of the dephasing time:

$$\mathrm{E}(\epsilon) \approx \frac{3}{4}\left(\frac{\tau}{4}\right)^2 \frac{2}{(T_2^*)^2} \tag{34}$$

$$\mathrm{E}(\epsilon) \approx \frac{3}{32}\left(\frac{\tau}{T_2^*}\right)^2 \tag{35}$$

Equation 35 gives us a direct method of predicting the fidelity of the CZ operation. Substituting in the average values given in table SII of $f_{\mathrm{Rabi}} = 171.0 \pm 1.1$ kHz and $T_2^* = 31.7 \pm 4.3$ μs, we get a theoretical error (and fidelity) of:

$$\mathrm{E}(\epsilon) = 0.32 \pm 0.09\% \tag{36}$$

$$\mathrm{E}(F) = 99.68 \pm 0.09\% \tag{37}$$

which is in agreement with the experimental average value of $F = 99.49 \pm 0.20$ %. While within uncertainties, the experimental fidelity could potentially include effects from miscalibration (ie. $E(\delta) \neq 0$), and also effects from dephasing of the nuclear spins during the application of the 2π ESR pulse. Despite this, Eq. 35 provides an upper bound on the qubit fidelity. Equation 35 also indicates quantities to optimise in order to increase two-qubit fidelities further, namely by aiming to extend electron T_2^* times and reduce electron gate times τ.

X. ADDITIONAL DATA FOR GROVER'S ALGORITHM

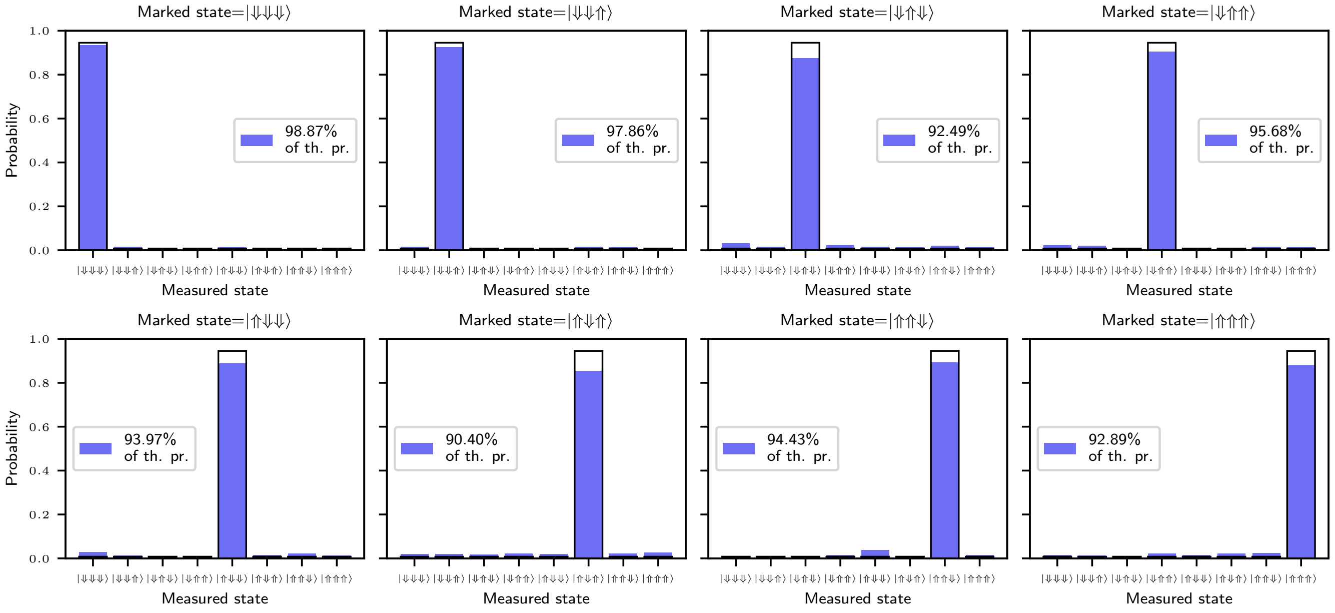

FIG. S5. **Grover's algorithm executed with all possible marked states.** For each measurement we indicate the probability of finding the correct marked state normalized by the theoretical maximum probability of finding the marked state with two Grover iterations (94.53%).

In Fig. 4b of the main text we show Grover's algorithm executed with $|\Downarrow\Downarrow\Downarrow\rangle$ as the marked state. We also ran this algorithm using all other possible states as the marked state, and the results are shown in Fig. S5. We achieve an average probability of $(89.40 \pm 2.49)\%$ of measuring the marked state, which corresponds to $(94.57 \pm 2.63)\%$ of the theoretical maximum value (94.53%).

XI. ERROR BUDGET FOR GROVER'S ALGORITHM

In this work, we perform Grover's search algorithm over all potential marked states m, as shown in Fig. 4 of the main text and supplementary information Fig. S5. On average, the success probability of our algorithm is $(94.57 \pm 2.63)\%$ of the ideal success probability. In Table SIII, we list the operations needed to implement the algorithm as per Fig. 4a of the main text, along with the number of times that operation is used. In addition, we include the error of each unique operation, as characterised in the main text (for single- and multi-qubit operations) and in supplementary information section II (for SPAM errors). By multiplying these fidelities together, according to multiplicities given by the number of applications of each operation, we obtain an estimate of the average fidelity of the algorithm as 95.89

% (also shown in Table SIII as "Total (no idle errors)"), matching closely with the average experimental performance of the algorithm.

Operation	Num. Occurences	Fidelity
SPAM (n1)	1	99.46%
SPAM (n2)	1	99.42%
SPAM (n3)	1	99.57%
Single-qubit gate (n1)	5	99.98%
Single-qubit gate (n2)	5	99.95%
Single-qubit gate (n3)	5	99.95%
Multi-qubit gate	4	99.49%
**Total (w/o idle errors)**	-	**95.89%**
n1 idle during $\pi/2$ rotations on n2 & n3	4	99.98%
n2 idle during $\pi/2$ rotations on n1 & n3	4	99.62%
n3 idle during $\pi/2$ rotations on n1 & n2	4	99.70%
**Total (w/ idle errors)**	-	**93.23%**

TABLE SIII. **Error budget for the implementation of Grover's search algorithm performed in this work.** The fidelities in the rightmost column for gate errors are as characterised in the main text of this work and supplementary section II. The "Total (w/o idle errors)" row is found by multiplying together the above fidelities, according to multiplicities given by the number of occurrences of each operation. The idle fidelities are calculated as per the formula $f \approx \exp\left(-\left(\tau/T_2^*\right)^2\right)$, outlined in this supplementary section. The total fidelity with idle errors is the product of these idling fidelities (according to their multiplicities) and the total fidelity without idle errors.

The error budget given in Table SIII without idling errors slightly overestimates the observed fidelity of 94.57 % measured in this work, likely because the single-qubit gate fidelities do not include idling errors incurred on the other nuclear qubits during the single-qubit operation. From supplementary section IV we know that the nuclear spins experience noticeable dephasing of $T_2^* = 1.26$ ms, 0.49 ms and 0.60 ms for nuclear spins n1, n2 and n3 respectively. During control of n1, for example, n2 and n3 will experience T_2^* dephasing according to the duration of the gate on n1. Note that such dephasing will not impact fidelity if n2 or n3 are in a z-basis state (such as $|\Downarrow\rangle$, as they are at the start of the algorithm); and similarly, dephasing will not affect fidelity at the end of the circuit immediately before the final measurement. For the remaining idle times, we can approximate the phase error from the formula $f \approx \exp\left(-\left(\tau/T_2^*\right)^2\right)$, where τ is the total time spent idling. Using this formula and the rabi frequencies given in supplementary section IV, we append additional errors to Table SIII. The overall total fidelity (including these idling errors) of 93.24 % is again close to the observed average of 94.57 %, but now underestimates the fidelity. Resolving the discrepancy between these two values is beyond the scope of this work, but suggests that the implementation of Grover's algorithm performed in this work is to some extent resilient to T_2^* errors, potentially due to the regularity of $\pi/2$ rotations on all 3 qubits creating partial refocusing of the nuclear spins.

To further characterise the errors incurred during idling, we perform a modified single-qubit randomised benchmarking experiment. For each randomised benchmarking variation, we generate 3 independent randomised benchmarking sequences, one for each of the nuclear spin qubits. These sequences are then converted to a sequence of physical gates, labelled $\{g_1^{n1}, g_2^{n1}, g_3^{n1}, \ldots, g_{m_1}^{n1}\}$ for nuclear spin 1, $\{g_1^{n2}, g_2^{n2}, \ldots, g_{m_2}^{n2}\}$ for nuclear spin 2, and likewise for nuclear spin 3. In the modified single-qubit randomised benchmarking circuit, we apply these gates sequentially over the three nuclear spins, ie in the order $g_1^{n1}, g_1^{n2}, g_1^{n3}, g_2^{n1}, g_2^{n2}, \ldots$ (sequentially doing physical gates to each qubit in turn). If all physical gates for one nuclear spin have been exhausted (possible if eg. $m_1 \neq m_2$), that nuclear spin will be skipped until all nuclear spins have completed their respective full sequences of gates. The results of this experiment are shown in Fig. S6, showing fidelities of (99.92 ± 0.01)%, (99.60 ± 0.02)%, and (99.67 ± 0.02)% for nuclear spins 1, 2 and 3 respectively. This is in close agreement with the idle fidelities shown in Table. SIII, again supporting the idea that nuclear fidelities can be described accurately with (standard) randomised benchmarking fidelities, along with T_2^* dephasing effects.

XII. STATE-OF-THE-ART IMPLEMENTATIONS OF GROVER'S ALGORITHM

Table SIV summarizes the state-of-the-art experimental implementations of Grover's algorithm reported to-date in the literature across multiple qubit platforms. Grover's algorithm has been demonstrated for up to 5 qubits. However,

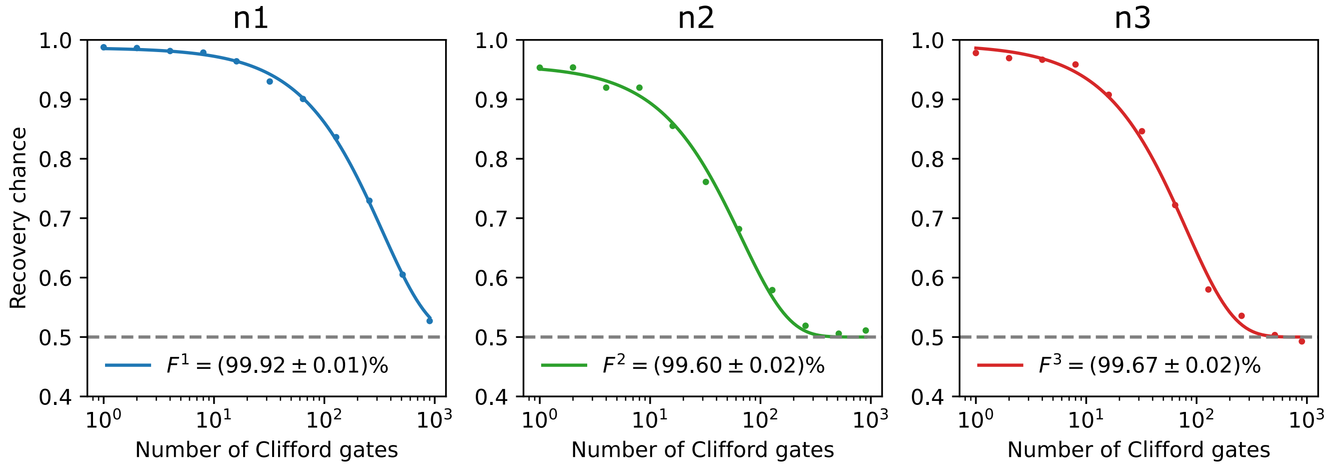

FIG. S6. **Modified single-qubit randomised benchmarking on the nuclear spins.** Here, randomised benchmarking is performed on all 3 nuclear spins in the same circuit, by performing physical gates on each of the nuclear spins sequentially (see text in this section for details). The reduced physical gate fidelities measured here (compared to randomised benchmarking performed individually on each nuclear spin, as shown in Fig. 1d of the main text) are modelled well by considering the extra idling time, and hence T_2^* dephasing, as calculated in Table SIII. The errors for the fidelities are obtained from the fits.

demonstrations on 4- and 5-qubit systems are limited in success probability ($< 50\%$) due to the long circuit lengths required. Our implementation with the marked $|{\Downarrow\Downarrow\Downarrow}\rangle$ state resulted in a success probability of 93.46% corresponding to 98.87% of the theoretical maximum, the highest reported to-date.

Reference	Year	Platform	Qubits	Iterations	Success prob.	Theoretical max.	Ratio
This paper ($|{\Downarrow\Downarrow\Downarrow}\rangle$ state)	2024	Si:P qubits (SQC)	3	2	93.46%	94.53%	0.9887
This paper (average)			3	2	89.40%	94.53%	0.9457
Hlembotskyi *et al.* [16]	2020	Ion trap qubits (Honeywell)	3	1	75.2%	78.1%	0.96
			5	1	18.7%	25.8%	0.72
Mandviwalla *et al.* [17]	2018	Superconducting qubits (IBM)	2	1	80.9%	100%	0.81
			3	2	59.7%	94.5%	0.63
			4	3	6.6%	96.1%	0.07
Zhang *et al.* [18]	2022	Ion trap qubits (Honeywell)	5	2	49%	60.2%	0.81
Zhang *et al.* [19]	2021	Superconducting qubits (IBM)	3	1	55.9%	78.1%	0.72
			3	2	63.8%	94.5%	0.68
			4	1	18.1%	47.3%	0.38
			4	2	19.5%	90.8%	0.21
			5	1	2.6%	25.8 %	0.10
Adedoyin *et al.* [20]	2018	Superconducting qubits (IBM)	2	1	65%	100 %	0.65
Friggatt *et al.* [21]	2017	Ion trap qubits	3	1	38.9%	78.1%	0.49
Gwinner *et al.* [22]	2020	Superconducting qubits (IBM)	4	1	21.0%	47.3 %	0.44
Stromberg *et al.* [23]	2018	Superconducting qubits (IBM)	4	1	6.6%	47.3 %	0.14
Watson *et al.* [11]	2018	Si/SiGe spin qubits	2	1	-	100 %	-

TABLE SIV. **State-of-the-art experimental implementations of Grover's algorithm in various qubit platforms.** The last column represents the ratio between the measured success probability and the theoretical maximum.

---

[1] X. Xue, M. Russ, N. Samkharadze, B. Undseth, A. Sammak, G. Scappucci, and L. M. K. Vandersypen, Quantum logic with spin qubits crossing the surface code threshold, Nature **601**, 343 (2022).

[2] A. Noiri, K. Takeda, T. Nakajima, T. Kobayashi, A. Sammak, G. Scappucci, and S. Tarucha, Fast universal quantum gate above the fault-tolerance threshold in silicon, Nature **601**, 338 (2022).

[3] K. Takeda, A. Noiri, T. Nakajima, T. Kobayashi, and S. Tarucha, Quantum error correction with silicon spin qubits, Nature **608**, 682 (2022).

[4] S. G. J. Philips, M. T. Mądzik, S. V. Amitonov, S. L. de Snoo, M. Russ, N. Kalhor, C. Volk, W. I. L. Lawrie, D. Brousse, L. Tryputen, B. P. Wuetz, A. Sammak, M. Veldhorst, G. Scappucci, and L. M. K. Vandersypen, Universal control of a six-qubit quantum processor in silicon, Nature **609**, 919 (2022).

[5] N. W. Hendrickx, W. I. L. Lawrie, M. Russ, F. van Riggelen, S. L. de Snoo, R. N. Schouten, A. Sammak, G. Scappucci, and M. Veldhorst, A four-qubit germanium quantum processor, Nature **591**, 580 (2021).

[6] F. van Riggelen, W. I. L. Lawrie, M. Russ, N. W. Hendrickx, A. Sammak, M. Rispler, B. M. Terhal, G. Scappucci, and M. Veldhorst, Phase flip code with semiconductor spin qubits, npj Quantum Information **8**, 124 (2022).

[7] M. T. Mądzik, S. Asaad, A. Youssry, B. Joecker, K. M. Rudinger, E. Nielsen, K. C. Young, T. J. Proctor, A. D. Baczewski, A. Laucht, V. Schmitt, F. E. Hudson, K. M. Itoh, A. M. Jakob, B. C. Johnson, D. N. Jamieson, A. S. Dzurak, C. Ferrie, R. Blume-Kohout, and A. Morello, Precision tomography of a three-qubit donor quantum processor in silicon, Nature **601**, 348 (2022).

[8] J. Reiner, Y. Chung, S. H. Misha, C. Lehner, C. Moehle, D. Poulos, S. Monir, K. J. Charde, P. Macha, L. Kranz, I. Thorvaldson, B. Thorgrimsson, D. Keith, Y. L. Hsueh, R. Rahman, S. K. Gorman, J. G. Keizer, and M. Y. Simmons, High-fidelity initialization and control of electron and nuclear spins in a four-qubit register, Nature Nanotechnology **19**, 605 (2024).

[9] G. Waldherr, Y. Wang, S. Zaiser, M. Jamali, T. Schulte-Herbrüggen, H. Abe, T. Ohshima, J. Isoya, J. F. Du, P. Neumann, and J. Wrachtrup, Quantum error correction in a solid-state hybrid spin register, Nature **506**, 204 (2014).

[10] J. T. Muhonen, A. Laucht, S. Simmons, J. P. Dehollain, R. Kalra, F. E. Hudson, S. Freer, K. M. Itoh, D. N. Jamieson, J. C. McCallum, A. S. Dzurak, and A. Morello, Quantifying the quantum gate fidelity of single-atom spin qubits in silicon by randomized benchmarking, Journal of Physics: Condensed Matter **27**, 154205 (2015).

[11] T. F. Watson, S. G. J. Philips, E. Kawakami, D. R. Ward, P. Scarlino, M. Veldhorst, D. E. Savage, M. G. Lagally, M. Friesen, S. N. Coppersmith, M. A. Eriksson, and L. M. K. Vandersypen, A programmable two-qubit quantum processor in silicon, Nature **555**, 633 (2018).

[12] W. Huang, C. H. Yang, K. W. Chan, T. Tanttu, B. Hensen, R. C. C. Leon, M. A. Fogarty, J. C. C. Hwang, F. E. Hudson, K. M. Itoh, A. Morello, A. Laucht, and A. S. Dzurak, Fidelity benchmarks for two-qubit gates in silicon, Nature **569**, 532 (2019).

[13] X. Xue, T. F. Watson, J. Helsen, D. R. Ward, D. E. Savage, M. G. Lagally, S. N. Coppersmith, M. A. Eriksson, S. Wehner, and L. M. K. Vandersypen, Benchmarking gate fidelities in a Si/SiGe two-qubit device, Phys. Rev. X **9**, 021011 (2019).

[14] M. Raginsky, A fidelity measure for quantum channels, Physics Letters A **290**, 11 (2001).

[15] O. E. Dial, M. D. Shulman, S. P. Harvey, H. Bluhm, V. Umansky, and A. Yacoby, Charge noise spectroscopy using coherent exchange oscillations in a singlet-triplet qubit, Phys. Rev. Lett. **110**, 146804 (2013).

[16] V. Hlembotskyi, R. Burczyński, W. Jarnicki, A. Szady, and J. Tułowiecki, Efficient unstructured search implementation on current ion-trap quantum processors, arXiv:2010.03841 (2020).

[17] A. Mandviwalla, K. Ohshiro, and B. Ji, Implementing grover's algorithm on the ibm quantum computers, in *2018 IEEE international conference on big data (big data)* (IEEE, 2018) pp. 2531–2537.

[18] K. Zhang, K. Yu, and V. Korepin, Quantum search on noisy intermediate-scale quantum devices, Europhysics Letters **140**, 18002 (2022).

[19] K. Zhang, P. Rao, K. Yu, H. Lim, and V. Korepin, Implementation of efficient quantum search algorithms on nisq computers, Quantum Information Processing **20**, 1 (2021).

[20] A. J., A. Adedoyin, J. Ambrosiano, P. Anisimov, W. Casper, G. Chennupati, C. Coffrin, H. Djidjev, D. Gunter, S. Karra, N. Lemons, S. Lin, A. Malyzhenkov, D. Mascarenas, S. Mniszewski, B. Nadiga, D. O'malley, D. Oyen, S. Pakin, L. Prasad, R. Roberts, P. Romero, N. Santhi, N. Sinitsyn, P. J. Swart, J. G. Wendelberger, B. Yoon, R. Zamora, W. Zhu, S. Eidenbenz, A. Bärtschi, P. J. Coles, M. Vuffray, and A. Y. Lokhov, Quantum algorithm implementations for beginners, ACM Transactions on Quantum Computing **3** (2022).

[21] C. Figgatt, D. Maslov, K. A. Landsman, N. M. Linke, S. Debnath, and C. Monroe, Complete 3-qubit grover search on a programmable quantum computer, Nature Communications **8**, 1918 (2017).

[22] J. Gwinner, M. Briański, W. Burkot, Ł. Czerwiński, and V. Hlembotskyi, Benchmarking 16-element quantum search algorithms on superconducting quantum processors, arXiv:2007.06539 (2020).

[23] P. Strömberg and V. Blomkvist Karlsson, *4-qubit Grover's algorithm implemented for the ibmqx5 architecture*, Ph.D. thesis, KTH (2018), available from: https://urn.kb.se/resolve?urn=urn:nbn:se:kth:diva-229797.
